# Expanding behavioral interventions through cancer warning labels in India: from cigarette packs to alcohol bottles

**DOI:** 10.3389/fpubh.2025.1641589

**Published:** 2025-07-24

**Authors:** Vaibhav Sahni, Deepak Saini, Abhishek Shankar

**Affiliations:** Department of Radiation Oncology, Dr. BR Ambedkar Institute Rotary Cancer Hospital, All India Institute of Medical Sciences, New Delhi, India

**Keywords:** cancer, alcohol, tobacco, warning label, public health

## Introduction

Among young adults and adolescents, disability-adjusted life years (DALYs) are contributed to most by burden attributable to alcohol, according to the Global Burden of Diseases, Injuries and Risk Factors Study (GBD) 2019 (1). Adolescence may be recognized as a critical time in the developmental stages of an individual wherein certain experiences can have long-term consequences. Adolescence forms a crucial period for the initiation and intensification of substance use behavior, including alcohol (2). Behavioral interventions instituted by means of alcohol warning labels may prove to be effective in affecting positive changes in the consumption habits of individuals belonging to this age group particularly in Low- and Middle-Income Countries (LMICs) where it is all the more important for certain sections of society to be educated and sensitized toward the consequences of substance use.

## The Southeast Asian perspective

The US Surgeon General, in January 2025, came out with an advisory regarding the consumption of alcohol and risk of cancer (3). This advisory stated that alcohol consumption demonstrably elevates the risk for developing at a minimum, seven types of cancer (colon/rectum, liver, breast, esophagus, larynx, pharynx and oral cavity) (3). The advisory also mentioned the mechanistic links between alcohol consumption and the risk of developing cancer along with the fact that this effect is observable regardless of gender (3).

Even before the release of this advisory, alcohol-attributable cancers have been recognized to contribute significantly to the global burden of disease (4).

Cancer warning labels on alcohol containers have been observed to be of benefit in reducing alcohol consumption and lowering the perception of consumption (5).

According to data from the National Family Health Survey (NFHS-4) alcohol consumption has been reported in 1% of the female and 29% of the male population in the 15–49 years age group (6).

Over the 1990–2017 period, Southeast Asian countries were noted to demonstrate the most significant elevation in per capita consumption of alcohol at 104% (Vietnam: 90%, India: 38%) (7). In fact, it is projected that the global increase in alcohol consumption may be largely driven by Southeast Asia (7). From the 2010 to 2017 period alone, the per capita consumption of alcohol increased by 34% in Southeast Asia which indicates that targeting consumption habits in LMICs forms a critical part of regional and overall global health (7).

## The Indian perspective

Cancer cases in India have seen a steep rise, with data from the 2012 to 2022 period suggesting a 36% uptick in incidence (1.01 million−1.38 million) (7).

GLOBOCAN 2022 data saw about 1.41 million new cancer cases in India with a 5-year prevalence at around 3.25 million and a total cancer mortality at 916,827 cases (8).

The alcohol attributable fraction for cancer and age standardized rate per 100,000 in India are 4.7% and 4.8, respectively, according to the GLOBOCAN 2020 data (9).

Data from 2016 suggested that 6.6% of Disease Adjusted Life Years in India were attributable to alcohol consumption which followed that of tobacco at 10.9% (10).

After the Parliament of India passed the Cigarettes and Other Tobacco Products Act in 2007, tobacco products were supposed to carry health warning labels which was delayed in its implementation till May 2009 and even then, till November 2011 the symbolic scorpion was used to convey warnings which was probably poorly understood (11). The warning labels underwent subsequent changes till April 2016 wherein these labels were mandated to cover a minimum of 85% (25% textual and 60% pictorial) of the packaging (11).

The Global Adult Tobacco Survey (GATS) in 2016–17 noted an increase by 16% for health warnings on cigarette packs, with pictorial health warnings demonstrating a 50% elevated impact on the intention to quit smoking cigarettes (12).

Warnings can be differentiated based upon the type of messaging involved into loss-framed and gain-framed which have an emphasis on associated risks/harms and the benefits of quitting, respectively (13). There is evidence in literature to suggest that gain-framed messaging possesses an advantage over loss-framed warnings but the research on such aspects has mainly focused on loss-framed warnings in the case of cigarette smoking (13). It is also suggested that a combination of messaging can help inform behavior change in a more effective manner which is based off the concept of the role individual beliefs play in determining outcomes (13).

India being an LMIC, this trend indicates the effect of cancer warning labels in modifying the behavior of a significant number of people consuming such products. LMICs may look into expanding the positive experience gained from tobacco warning labels to those pertaining to alcohol containers which clearly state a cancer risk from consumption. It may also be useful for these cancer labels to state that there is no lower threshold for alcohol-related cancer risk (14) along with the types of cancers demonstrably attributable to alcohol consumption so far.

For example, it has been observed that a number of people do tend to relate alcohol consumption with liver cancer but this relation with other types such as colon and breast cancer is not as well known (14, 15).

## Discussion

A crucial but often ignored aspect while considering warning labels is the multiplicative interaction of smoking and alcohol consumption in determining cancer risk. A National Cancer Institute (NCI) Workshop in December 2020 emphasized the importance of addressing the combined usage of tobacco and alcohol (16). Co-use of tobacco and alcohol has been found to be associated with a multiplicative effect in cancer risk, particularly for pharyngeal and oral sites (16). The importance of reciprocative warning labels on tobacco and alcohol product packaging is further underscored by the fact that alcohol usage has been observed to increase with an increase in cigarette smoking, with the former being associated with lower rates of quitting and higher relapse rates in smokers (16). It may also be worth considering to have helpful or constructive labeling on containers which guide the user to seek medical advice or undergo screening for cancer instead of being terminalistic in its messaging by suggesting graphic or fatal outcomes upon consumption.

**Figure 1:**
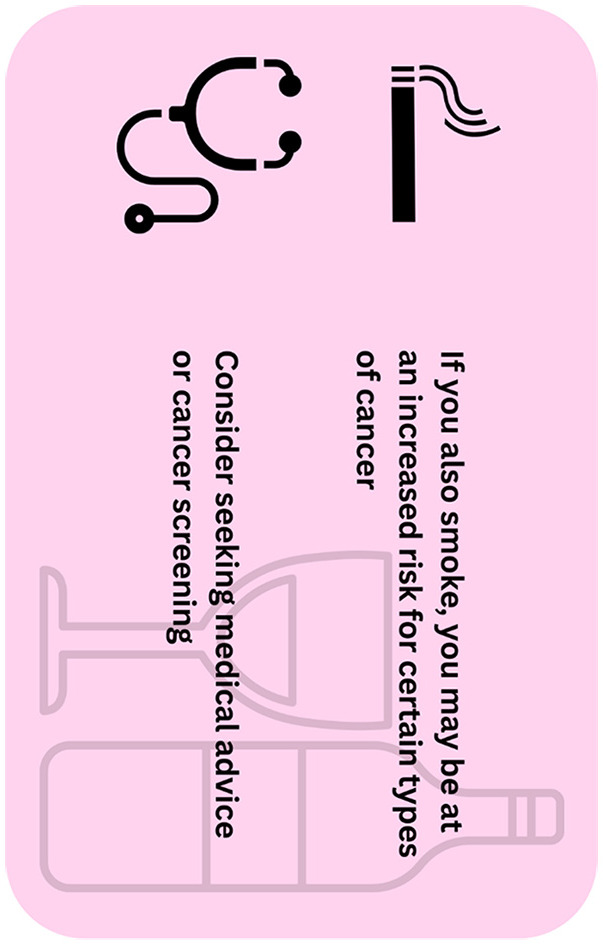
Example of a reciprocal warning label with constructive wording.

Since cancer as a disease may present as a result of the combined effect of alcohol and tobacco consumption, it makes sense to place such cancer warning labels and not address these risk factors in isolation. Figure 1 provides the example of one such cancer warning label which might be used on alcohol bottles.

India is developing rapidly and at the same time has reportedly the greatest increase in per capita alcohol consumption in the Southeast Asia region (2).

Existing policy frameworks in India have suffered from a dearth of consensus, fragmented implementation and economic influences which has resulted in a less than desirable outcome in terms of reducing harm from the consumption of alcohol (2). Vastly, the focus of existing policies tends to be on adult alcohol dependence and does not incorporate a perspective on preventive strategies (2). With the largest population of adolescents in the world residing in India, forming 21% of its own populace, reports of individuals as young as 13–15 years taking up alcohol consumption are concerning to say the least (2).

It is imperative to consider preventive strategies as the ones mentioned in this paper in order to save an entire generation from negative health, social and economic life experiences.

## References

[1] World Health Organization. Global Status Report on Alcohol and Health-2018. Geneva: WHO (2018).

[2] Nadkarni A Tu A Garg A Gupta D Gupta S Bhatia U. Alcohol use among adolescents in India: a systematic review. Glob Ment Health. (2022) 9:1–25. 10.1017/gmh.2021.48 36618747 PMC9806994

[3] US Department of Health and Human Services. Alcohol and cancer risk, 2025. The US Surgeon General's Advisory. Available at: https://www.hhs.gov/surgeongeneral/reports-and-publications/alcohol-cancer/index.html (Accessed March 18, 2025)

[4] Rumgay H Shield K Charvat H Ferrari P Sornpaisarn B Obot I. Global burden of cancer in 2020 attributable to alcohol consumption: a population-based study. Lancet Oncol. (2021) 22:1071–80. 10.1016/S1470-2045(21)00279-5 34270924 PMC8324483

[5] Joyce KM Davidson M Manly E Stewart SH Al-Hamdani M. A systematic review on the impact of alcohol warning labels. J Addict Dis. (2024) 42:170–93. 10.1080/10550887.2023.2210020 37212771

[6] Gowri SM Belavendra A Vasan SK Keerthi S Andreasson S. Prevalence and long-term change in alcohol consumption: results from a population-based cohort in Southern India. Int J Ment Health Syst. (2024) 18:30. 10.1186/s13033-024-00650-w 39390493 PMC11465489

[7] Singh K Grover A Dhanasekaran K. Unveiling the cancer epidemic in India: a glimpse into GLOBOCAN 2022 and past patterns. Lancet Reg Health Southeast Asia. (2025) 34:100546. 10.1016/j.lansea.2025.100546 40070552 PMC11893321

[8] IARC WHO. India Fact Sheet. Available at: https://gco.iarc.who.int/media/globocan/factsheets/populations/356-india-fact-sheet.pdf (Accessed June 22, 2025)

[9] WHO IARC. Cancers Attributable to Alcohol. Available at: https://gco.iarc.fr/causes/alcohol/tools-map?mode=1&sex=0&population=1&continent=0&cancer=40&key=paf&age_group=3 (Accessed June 18, 2025).

[10] Mehrotra R Kapahtia S Kaur T Priyanka KY Dhaliwal RS. Alcohol & cancer: Evidence to action. Indian J Med Res. (2022) 155:227–31. 10.4103/ijmr.IJMR_4037_20 35946199 PMC9629523

[11] Holdroyd I Puntambekar N Driezen P Gravely S Quah ACK Xu SS. Evaluation of the effectiveness of the Indian government's policies to strengthen health warning labels on smokeless tobacco products: findings from the 2010-2019 Tobacco Control Project India Surveys. Tob Control. (2024) 30:tc-2023-058281. 10.1136/tc-2023-058281 38216314 PMC11239794

[12] Tripathy JP Verma M. Impact of health warning labels on cigarette packs in India: findings from the global adult tobacco survey 2016-17. Behav Med. (2022) 48:171–80. 10.1080/08964289.2020.1796571 32703087

[13] Mays D Turner MM Zhao X Evans WD Luta G Tercyak KP. Framing pictorial cigarette warning labels to motivate young smokers to quit. Nicotine Tob Res. (2015) 17:769–75. 10.1093/ntr/ntu164 25143295 PMC4542675

[14] Ferreira-Borges C Kokole D Galea G Neufeld M Rehm J. Labels warning about alcohol-attributable cancer risks should be mandated urgently. Lancet Public Health. (2025) 13:S2468-2667(25)00040-4. 10.1016/S2468-2667(25)00040-4 39956133

[15] Kokole D Ferreira-Borges C Galea G Tran A Rehm J Neufeld M. Public awareness of the alcohol-cancer link in the EU and UK: a scoping review. Eur J Public Health. (2023) 33:1128–47. 10.1093/eurpub/ckad141 37802887 PMC10710347

[16] National Cancer Institute. Co-Use of Tobacco with Alcohol and Cannabis. Available at: https://cancercontrol.cancer.gov/brp/tcrb/co-use-tobacco-alcohol-cannabis (Accessed March 15, 2025)

